# The Perception of Occupational Safety and Health (OSH) Regulation and Innovation Efficiency in the Construction Industry: Evidence from South Korea

**DOI:** 10.3390/ijerph18052334

**Published:** 2021-02-27

**Authors:** Jaeho Shin, Yeongjun Kim, Changhee Kim

**Affiliations:** 1 College of Social Science, Hansung University, Seoul 02876, Korea; jhshin@hansung.ac.kr; 2 College of Business Administration, Incheon National University, Incheon 22012, Korea; brent.kim@inu.ac.kr

**Keywords:** occupational safety and health regulation, OSH, perception of regulations, innovation efficiency, construction industry

## Abstract

Due to safety issues in the construction industry, interest in research on occupational safety and health (OSH) regulations remains high. Previous studies indicated that OSH regulations not only affect performance in and of themselves, but also indirectly by increasing awareness of such regulations. Studies also demonstrated that OSH regulation can affect innovation and corporate safety. However, the effect of OSH regulation on innovation remains unclear, as the relationship between the perception of OSH regulation and innovation is not fully understood. This study measures the innovation efficiency of companies in the Korean construction industry using data envelopment analysis (DEA), and investigates the relationship between innovation efficiency and companies’ perceptions of OSH regulations. Results indicate that companies that positively recognize OSH regulations tend to be more innovative than those that do not. This study also validates differences in innovation efficiency depending on the perception of OSH regulations by bootstrap DEA. The results of this study suggest appropriate strategies to promote innovation in the construction industry from the perspectives of both government and practitioners in firms.

## 1. Introduction

**Figure 1:**
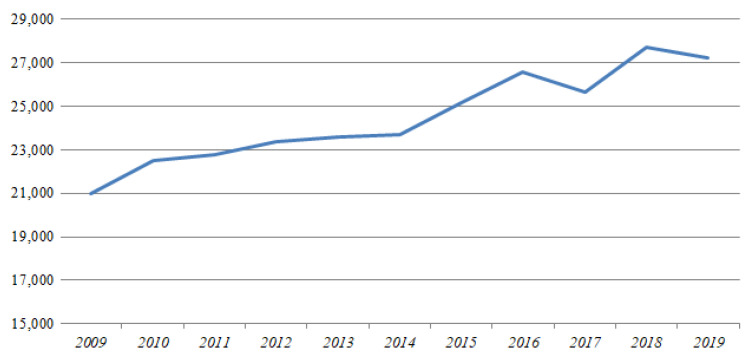
Casualties in the construction industry in South Korea [4].

According to the International Labour Organization [1], the number of workers dying from workplace accidents and work-related diseases each year is estimated to reach 2.78 million, with 374 million additional workers suffering from nonfatal occupational accidents. Such accidents are particularly prevalent in the construction industry, due to its unique characteristics [2]. South Korean government-established safety-management tasks, construction-site safety management, and the assessment of safety management at all stages of construction are based on guidelines for safety management in construction projects and safety-management manuals distributed to all participants in construction projects [3]. Despite minor fluctuations, the number of casualties in the construction industry in Korea is increasing over time, with 27,211 injuries and 517 deaths as of 2019 (see Figure 1) based on data from the Korean Ministry of Employment and Labor [4]. The construction industry accounts for 27% of casualties and deaths in all industries, indicating the importance of safety regulations [4]. According to the Korean Ministry of Land, Infrastructure, and Transport [3], vulnerable areas such as small private sites still exist in blind spots of safety management, where safety issues are caused by the indifference of construction executives. Such safety issues in the construction industry are not limited to South Korea, but are globally recognized as serious problems [5,6,7,8].

As the need for the introduction of occupational safety and health (OSH) regulations was addressed, research on the impact of OSH regulations was also conducted [9,10,11,12,13]. OSH regulations can have significant impact on innovation and the safety of a company, and firm performance is influenced by both the regulations themselves, and their perception and attitudes towards them [14,15,16]. Accordingly, in order to introduce appropriate OSH regulations, the impact of occupational safety regulations on safety and innovation should be considered. It is also important to consider the impact of a company’s attitude towards such regulations on overall firm performance.

Nevertheless, the impact of the perception of such regulations on innovation remains poorly understood. By utilizing measurements of innovative efficiency, this work aims to determine how innovation efficiency differs depending on a company’s perception of OSH regulation. This study also aims to reveal an unknown link between OSH regulations and firm performance. Lastly, we suggest appropriate OSH strategies to enhance innovation in both government and firms.

## 2. Theoretical Background

### 2.1. OSH, Performance, and Innovation

Research on the impact of occupational safety and health regulations on the construction industry is ongoing. The introduction of OSH regulations was demonstrated to reduce accidents [10]. The advantages of introducing OSH regulations also include positive effects on productivity, corporate performance, and future income [11,12,13].

However, while appropriate OSH regulations in the construction industry enhance safety, excessive regulations may have negative impact [9]. Such regulations may not effectively reduce accidents in the construction industry by themselves, and should be accompanied by additional strategies to ensure that workers meet safety standards [17].

OSH regulations have significant impact on innovation, safety, and corporate performance. As such, the effect of industrial safety on the climate for innovation should be considered when designing regulations [18]. Moreover, striving for sustainability and safety can be sources of innovation [19,20], and investment in OSH can thus result in a climate for innovation [14,15]. Safety in the construction industry may be improved through the use of innovations such as 3D printing, robots, and drones [21,22,23].

The research literature indicates that OSH regulations not only affect safety, but also significantly affect innovation. Nevertheless, empirical evidence for the relationship between OSH regulation and innovation is still insufficient [14,15].

### 2.2. Perception of OSH and Performance

OSH systems themselves do not have a positive effect on safety, and can only be effective when positive responses to safety culture in the workplace are maintained [16]. The positive effects of OSH regulation are seen when activities intended to improve awareness, such as safety-culture campaigns, occur constantly [17]. Safety culture encompasses psychological (how people feel), behavioral (what people do), and situational (what an organization has) aspects. Psychological aspects are also referred to as the safety climate [24,25].

To form a strong safety culture, several factors must be involved, of which the employer’s leadership is key [16,26]. This is because the company’s will to create a safe environment is directly related to workers’ perceptions of safety climate, which fosters workers’ safe work behavior, reduces the frequency of accidents, and supports workers’ general welfare and motivation [27,28,29]. Workers recognize the firm’s commitment to safety as company support, and reward it with greater commitment, participation, and loyalty [30,31].

That is, the employer’s will is a prerequisite for creating a positive safety climate, as workers try to comply with industrial safety regulations, and follow other safety recommendations when managers demonstrate their commitment to and support for safety [32]. Therefore, for the effect of OSH regulations to be positively realized, an explicit culture of safety should be instilled throughout companies on the basis of employer leadership. Recent studies drew attention to the lack of interest in OSH and encouraged efforts to improve OSH awareness [33,34].

The recognition of OSH regulations not only has a significant impact on safety, including a reduction in accidents [27,28,29], but also on corporate performance, by lowering employees’ withdrawal behavior, and increasing affective commitment and job satisfaction [30,31]. Despite the fact that OSH regulations can affect innovation [14,15,18,19,20], the impact of the perception of regulation on innovation is still unknown.

### 2.3. Innovation and Innovation Efficiency

Innovation efficiency refers to the ability to convert inputs into outputs. Since the output of innovation is not guaranteed, even when a specific amount of input is expended, the assessment of innovation should be measured with innovation efficiency rather than innovation performance itself [35]. Accordingly, research on innovation measures its efficiency, and recent studies on innovation efficiency are compared in Table 1.

In summary, OSH regulations have significant impact on innovation and safety. In addition, OSH regulations both directly and indirectly affect firm performance by shaping an enterprise’s attitude toward these regulations. Accordingly, prior research regarding the direct impact of OSH regulations on innovation, in addition to the impact of the perception of industrial safety regulations on safety, was discussed. However, the relationship between OSH regulations and innovation remains unclear, as the effect of the perception of OSH regulations on innovation is not fully understood.

Therefore, this research verifies how innovation differs depending on the perception of OSH regulations by measuring innovation efficiency. This study provides a basis for establishing appropriate regulations from the perspective of government, and provides suggestions for improving innovation efficiency in firms.

## 3. Research Methods

### 3.1. Research Flow

**Figure 2:**
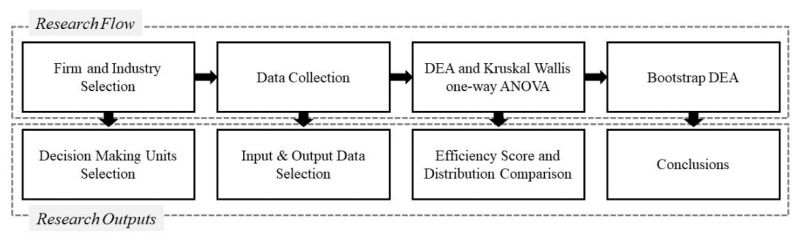
Research flowchart.

This study was conducted according to the research flowchart in Figure 2. First, input-oriented BCC data envelopment analysis (DEA) was implemented to measure the innovation efficiencies of construction-industry enterprises. Next, the study classified enterprises into four groups on the basis of their perception of OSH regulations (Group 1, OSH regulations perceived as significantly promoting innovation; Group 2, OSH regulations somewhat perceived as promoting innovation; Group 3, OSH regulations not perceived as impacting innovation; and Group 4, OSH regulations somewhat perceived as negatively impacting innovation). Kruskal–Wallis one-way ANOVA was performed to verify differences in the distribution of innovative efficiency among different groups. Bootstrap DEA was utilized to overcome the limitations of traditional DEA and derive bootstrapped innovation efficiency. Lastly, the averages of bootstrapped efficiencies between groups were compared.

### 3.2. Data Envelopment Analysis (DEA) and Bootstrap DEA

Typical techniques for measuring efficiency include stochastic frontier analysis (SFA) and data envelopment analysis (DEA); the former is parametric, while the latter is a nonparametric method [45]. SFA measures efficiency by estimating the frontier function with a quantitative econometric method, while DEA is a technique for evaluating the relative efficiency of comparable decision-making units (DMUs) [46]. In general, however, performance measurements of multiple input and output production systems cannot be described in the form of specific functions [47]. On the other hand, the nonparametric characteristics of DEA not only allow for multiple inputs and outputs to be used regardless of measurement units, but also do not require prior information on the basic functional form and weight [46,47,48]. Therefore, DEA is particularly suitable for measuring the performance of production systems with multiple inputs and outputs [49,50,51].

Due to these advantages, DEA is widely used in innovative research [46,48]. This is because innovative activities are complex and multidimensional processes consisting of interactions between various inputs and outputs. Therefore, the ability to innovate cannot be measured on a single-dimensional scale [46,47,48,52,53,54,55]. 

**Figure 3:**
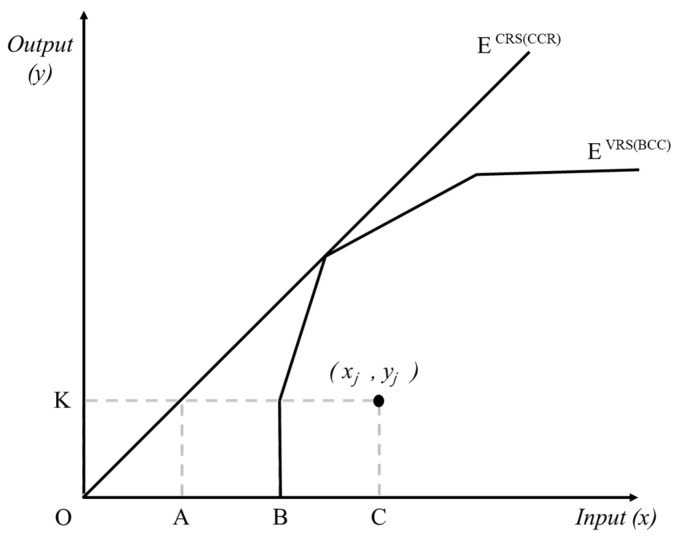
Frontiers of BCC and CCR models.

In this work, the innovation efficiencies of companies in the construction industry were evaluated by adopting an input-oriented BCC DEA model proposed by Banker et al. [56]. Differences between BCC and CCR DEA models are shown in Table 2 and Figure 3, and the equation of the BCC model is provided below in Equation (1). After BCC model analysis, Kruskal–Wallis one-way ANOVA was performed to verify the difference in the distribution of innovative efficiency depending on the companies’ perceptions of OSH regulations. Kruskal–Wallis one-way ANOVA is a nonparametric technique for measuring significant differences in continuous variables among three or more groups, which is suitable for measuring differences in efficiency scores evaluated with DEA [57].

$$Minimize\;\theta_{0}\;\left(Efficiency\;of\;DMU_{0}\right)\;subject\;to.\;\overset{l}{\underset{j=1}{\sum }}x_{ij}\lambda \leq \theta_{0}x_{i0}\;i\;=\;1,\;2,\ldots ,l$$

(1) $$\overset{m}{\underset{j=1}{\sum }}y_{rj}\lambda_{j}\geq y_{r0}\;r\;=\;1,\;2,\ldots ,\;m\;\overset{n}{\underset{j=1}{\sum }}\lambda_{j}=1\;\left(\lambda_{j}\geq 0\right)\;j\;=\;1\;,\;2,\ldots ,\;n$$

**Figure 4:**
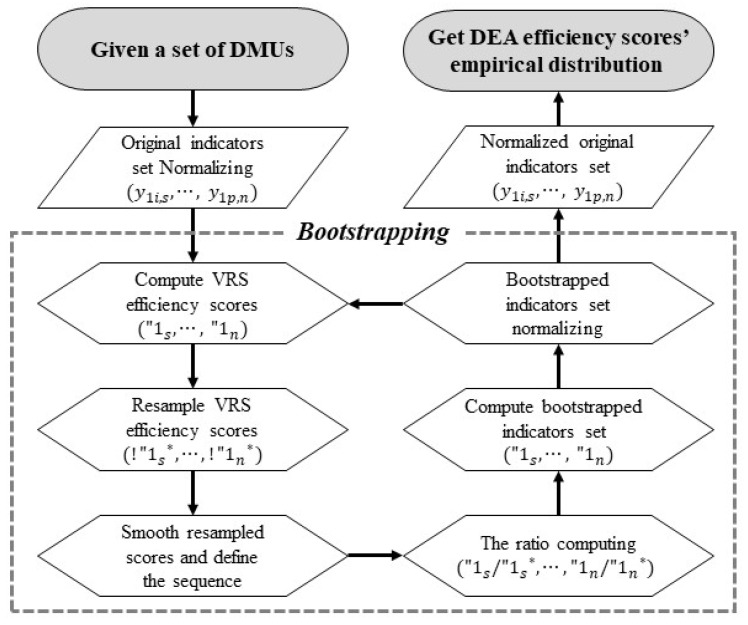
Bootstrap procedure based on [58,59,60].

However, DEA can produce errors when estimating the average or standard deviation of relative efficiency due to its nonparametric characteristics, and limitations exist, such as the method’s failure to provide information on the uncertainty of estimates [58,59]. To overcome this limitation and compare the averages of efficiency, this study implemented bootstrap DEA. The bootstrap procedure was repeated 2000 times to ensure a suitable confidence interval, as suggested by Simar and Wilson [58], and the confidence interval was estimated following Kneip et al. [60]. The bootstrap procedure is elaborated in Figure 4.

### 3.3. Variable Selection and Data

The DEA evaluates relative efficiency by measuring the relative distance of each decision-making unit from the efficient frontier derived from inputs and outputs [49]. Therefore, input and output selection is one of the most important parts in performing DEA and should be carried out carefully following a sufficient literature review [61,62].

**Figure 5:**
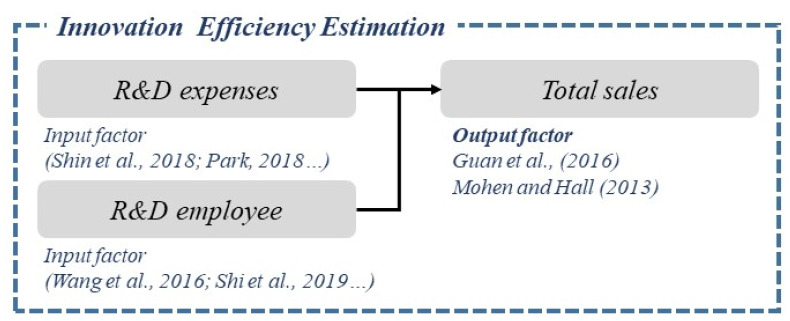
Input and output for efficiency measurement.

As illustrated in Table 1, diverse studies on innovation efficiency adopt innovation costs and R&D personnel as inputs, and sales as outputs [36,37,38,39,40,41,42,43,44]. This study captures innovation costs and R&D personnel as inputs, and total sales as the output, as in previous studies (see Figure 5).

Data regarding the construction industry from the 2018 Korea Innovation Survey was utilized. As the samples of the survey were enterprises, each sample represents a different company. The CEO or executive officer was asked to respond, and where this was not possible, a working-level staff member responded on the basis of objective evidence. The survey sought overall information on innovation over the past three years (2015–2017). Since the study measured input and output factors on the basis of data from 2017, the efficiency values represent innovation efficiency in 2017. Conversely, the perception of impact of OSH regulation, an environmental variable, asked about perception over the period of 2015–2017, not 2017 alone. The questionnaire on the perception of OSH regulation is presented in Appendix A. In this study, only companies that conducted innovation activities were selected as samples to secure the homogeneity of DMUs, and 90 construction companies out of 220 were utilized in actual analysis. This number satisfied the criteria of the recommended number of DMUs suggested by Boussofiane et al. [63], and Banker et al. [56].

This study classified companies on the basis of their attitudes towards OSH regulations, as the purpose of the study was to verify differences in innovation efficiency depending on their attitudes. The effect of the perception of OSH regulation was measured on a five-point Likert scale, with lower numbers being positive and higher numbers being negative. Descriptive statistics are shown in Table 3.

## 4. Results

The results of efficiency estimation are shown in Table 4, and pairwise comparison results from Kruskal–Wallis one-way ANOVA are shown in Table 5.

**Figure 6:**
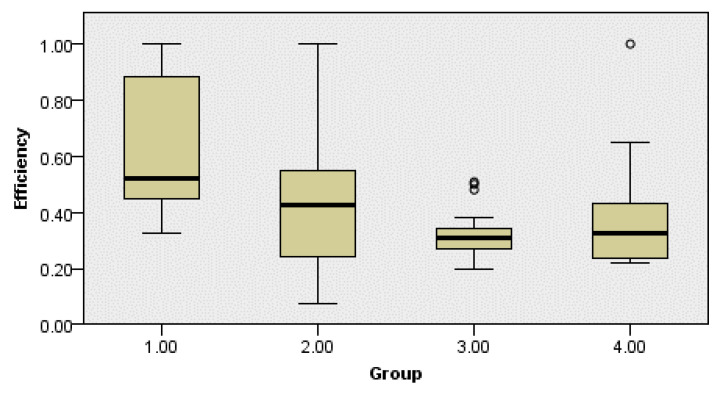
Box and whisker plot of VRS efficiency by groups.

Table 5 shows that the significant difference in the efficiency between Group 1 (perceive OSH regulation as significantly improving innovation) and Group 3 (perceive OSH regulation as having no impact on innovation) was verified. The box and whisker plot of innovation-efficiency distributions among groups clarifies the differences (see Figure 6). The median of the efficiency of Group 1 (0.522) was higher than that of other groups (Group 2 = 0.424; Group 3 = 0.309, Group 4 = 0.327). Compared to Group 3 (perceive OSH regulation as having no effect on innovation), Groups 1 and 2 (perceive OSH regulation as impacting positively on innovation) showed higher innovation efficiency, while Group 4 (perceive OSH regulation as having a somewhat negative effect on innovation) showed little difference. 

**Figure 7:**
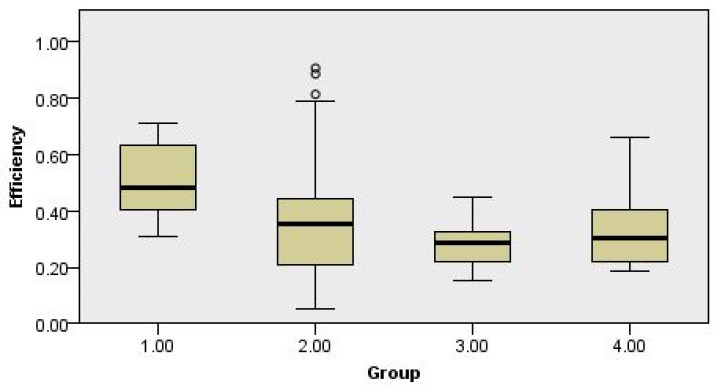
Box and whisker plot of bootstrap efficiency by groups.

The results of bootstrap DEA are shown in Table 6, and the box and whisker plot of the distribution of bootstrap efficiency is presented in Figure 7. Bootstrap DEA results are in accordance with the results of input-orientated BCC DEA. The average innovation efficiency of Group 3 was 0.2870, of Group 2 was 0.3640, and Group 1 was 0.5108, which supports the hypothesis that the more companies positively recognize the impact of OSH regulation, the higher their innovation efficiency is. The average efficiency of Group 4 is 0.3447, a difference of only 0.0577 from Group 3. The comparison among groups was also conducted with single-factor ANOVA, and results are shown in Table 7. As the F value (2.8637) was greater than the F critical value (2.7106), and the *p* value (0.0413) was less than the significance level of 0.05, the null hypothesis was rejected (the four group means were not all equal).

## 5. Conclusions

### 5.1. Discussion

According to Hudson [64], a firm’s safety culture generally begins with the introduction of occupational safety and health management systems (OSHMS) by companies according to the government’s OSH regulation, and the extent of safety-culture settlement evolves through five sequential stages: pathological, reactive, calculative, proactive, and generative. However, excessive OSH regulation hinders the growth of the company’s safety culture [64,65] and has other potential adverse effects [9]. Accordingly, recent OSH regulations are shifting away from compulsory legislation and regulation to inducing companies to voluntarily introduce OSHMS [65] while emphasizing the role of government as facilitator rather than regulator [64].

The role of the Korean government is also gradually shifting from regulator to facilitator. In Korea, OSH regulations are established on the basis of industrial-accident prevention plans. The first industrial-accident prevention plan (2000–2004) included extensive guidance and support for sectors vulnerable to industrial accidents in its major agenda. The second industrial-accident prevention plan (2005–2009) included the overall agenda of the first while reinforcing the responsibility of workplaces. Despite this, the government-led project did not establish any internal systems, resulting in a high accident rate that fell short of its original target: 0.85% in 2004 (the last year of the first industrial-accident prevention plan), and 0.7% in 2009 (the last year of the second industrial-accident prevention plan).

On the other hand, the third (2010–2014) and fourth (2015–2019) industrial-accident prevention plans broke away from government-led top–down policy-delivery systems, and instead pursued decentralization and diversification to reflect on-site demand, change workers’ perceptions of OSH, and focus on establishing autonomous OSHMS by business owners and workers. In addition, the government established autonomous industrial-accident prevention activities and internalized safety awareness through the spread of safety culture as major policy goals. As a result, the accident rate was 0.53% in 2014 (the last year of the third industrial-accident prevention plan) and 0.58% in 2019 (the last year of the fourth industrial accident-prevention plan), significantly improving safety [3].

Although the role of the Korean government in OSH regulation was never defined, it gradually shifted from regulator to facilitator during the industrial-accident prevention plans. More recently, the government has played a role in helping companies to voluntarily establish safety culture and a positive perception of OSH, which results in safety improvements. This is consistent with the findings of previous studies that positive perceptions of OSH regulations have positive effects on safety [27,28,29,30,31].

The perceptions of OSH regulations can also affect innovation [14,15,18,19,20]. Thus, the impact of OSH regulation awareness on innovation may also vary depending on whether the government’s role in OSH regulation is as regulator or facilitator. Results demonstrate that, the more positive the perception of OSH regulation is, the more efficient innovation is, but the data utilized in this study correspond to the years of 2015–2017 (the fourth industrial-accident prevention plan period). Therefore, for a deeper understanding of the relationship between OSH regulation awareness and innovation, research on the influence of the perception of OSH regulation on innovation is required when the government assumes the role of regulator.

### 5.2. Implications, Limitations, and Suggestions for Future Research

This study has the following academic implications: first, this work verified the impact of the perception of OSH regulation on innovation efficiency, expanding the discussion of prior studies on the impact of OSH regulation on innovation, and the impact of the perception of OSH regulation on firm performance. Furthermore, this study measured innovation efficiency instead of innovation performance, and extended the discussion of the impact of perception of OSH regulation on innovation performance. Therefore, this study demonstrates the previously undiscovered relationship between OSH regulation and innovation.

This study has the following practical implications as well: first, given that innovation efficiency may increase when firms positively perceive OSH regulation, firms could increase innovation by promoting a safety climate. It is important for practitioners to positively recognize OSH regulations and to accept regulations as a factor with positive impact, rather than an obstacle to innovation. In addition, from the perspective of the government, it is necessary to endeavor to correct attitudes towards OSH regulations, and to establish and apply regulations to encourage innovation in the construction industry.

Despite the implications, several limitations must be acknowledged. First, though the study verified differences in innovation efficiency depending on the perception of OSH regulation, it could not examine the overall relationship among OSH regulation, regulation perception, and innovation efficiency. In addition, while other studies utilized the sales of innovative products and the number of patent applications as output factors, this study considered total sales alone.

Such limitations are due to the data utilized in this study. The data were provided by managers of firms and reflected the perception of these managers on OSH regulation without information on the regulations themselves. In addition, companies could not accurately measure the revenue of innovation products; therefore, other factors could not be considered. Though innovation affects overall sales, it is required to use other output factors, such as the sales of innovative products and the number of patent applications, in order to more accurately measure the efficiency of innovation. Therefore, a future study could apply qualitative techniques such as interviews and utilize additional outputs of innovation to make meaningful conclusions about OSH regulations.

## Figures and Tables

**Table 1:** Recent studies on innovation efficiency.

Source	Method	Decision-Making Units (DMUs)	Input Factors	Output Factors
Żółtaszek and Olejnik (2021) [36]	DEA, Malmquist index	28 European states and 261 NUTS 2 regions	(1) Human capital (2) R&D expenditure	(1) Number of patents (2) GDP
Wang et al. (2021) [37]	Two-stage StoNED (stochastic non-parametric envelopment of data) model	45 Chinese civil–military integration enterprises	(1) Proportion of R&D personnel (2) Proportion of R&D investment to operating income	(1) Number of patent applications (2) Operating income
Chen et al. (2021) [38]	SBM (slacks-based measure) model-based network DEA, Malmquist index	16 Chinese new-energy vehicle enterprises	(1) R&D investment (2) Full-time equivalent R&D investment personnel	(1) Main business income (2) Operating profit
Zeng et al. (2021) [39]	Super-SBM (slacks-based measure) DEA	30 Chinese provinces	(1) R&D personnel (2) R&D capital stock (3) Total energy consumption	(1) Number of patent applications (2) New product sales revenue (3) Environmental pollution
Song and Zhang (2020) [40]	Window DEA	49 Chinese energy companies	(1) R&D personnel (2) R&D expenditure (3) Fixed asset balance at end of year	(1) Annual operating revenue (2) Patent quality (3) Annual public-welfare donation (4) Positive environmental governance program
Xu et al. (2020) [41]	Superefficiency SBM model	30 Chinese provinces	(1) R&D personnel full-time equivalent (2) R&D expenditure (3) New product development projects	(1) Invention applications (2) New product sales (3) SO_2_ emissions (4) CO_2_ emissions
Wang et al. (2020) [42]	Network DEA	18 Chinese high-tech industries	(1) R&D personnel (2) R&D expenditure (3) Technical transformation expenditure (4) Newly increased fixed assets	(1) Patent applications (2) Sales revenue of new products
Min et al. (2020) [43]	Network DEA	16 regions in Korea	(1) R&D expenses (2) R&D personnel	(1) Patents (2) Scientific publications (3) Rate of technology transfer (4) Export value (5) Gross value added
Kim and Shin (2019) [44]	DEA	72 Korean logistics firms	(1) Number of employees (2) Innovation expenses	(1) Sales

**Table 2:** Comparison of DEA models.

DEA Model Selection		Return to Scale	
		Constant Return to Scale (CRS)	Variable Return to Scale (VRS)
Controllable Factor	Input Factor	Input-oriented CCR DEA Model	Input-oriented BCC DEA Model
	Output Factor	Output-oriented CCR DEA Model	Output-oriented BCC DEA Model

**Table 3:** Descriptive statistics.

Factors		Max	Min	Mean	SD
Input	R&D expenses	7500.00	73.00	750.04	1104.23
	R&D employees	517	1	23	69
Output	Sales	331,695.00	566.00	25,392.78	54,566.10
Perception of OSH regulation		4.00	1.00	2.33	0.77

**Table 4:** Frequency table of BCC DEA results.

Score	Frequency	Percentage	Score	Frequency	Percentage
0.0–0.1	4	4.4	0.5–0.6	9	10.0
0.1–0.2	5	5.6	0.6–0.7	5	5.6
0.2–0.3	22	24.4	0.7–0.8	1	1.1
0.3–0.4	15	16.7	0.8–0.9	1	1.1
0.4–0.5	17	18.9	0.9–1.0	11	12.2

**Table 5:** Pairwise comparison results.

Comparison	Test Statistics	Std. Error	Std. Test Statistic	Sig. Test Statistic
C1 (G1–G2)	19.321	10.464	1.847	0.389
C2 (G1–G3)	32.109	11.721	2.739	0.037 **
C3 (G1–G4)	24.286	12.863	1.888	0.354
C4 (G2–G3)	12.788	7.228	1.769	0.461
C5 (G2–G4)	4.964	8.960	0.554	1.000
C6 (G3–G4)	−7.824	10.402	−0.752	1.000

** *p* < 0.05.

**Table 6:** Frequency table of bootstrap DEA results.

Score	Frequency	Percent	Score	Frequency	Percent
0.0–0.1	5	5.6	0.5–0.6	5	5.6
0.1–0.2	11	12.2	0.6–0.7	3	3.3
0.2–0.3	21	23.3	0.7–0.8	2	2.2
0.3–0.4	20	22.2	0.8–0.9	2	2.2
0.4–0.5	20	22.2	0.9–1.0	1	1.1

**Table 7:** ANOVA results of bootstrap DEA scores by groups.

Source of Variation	*SS*	*df*	*MS*	*F*	*p Value*	*F Crit*
Between groups	0.2529	3	0.0843	2.8637	0.0413 **	2.7106
Within groups	2.5313	86	0.0294			
Total	2.7842	89				

** *p* < 0.05.

## Data Availability

Data are available from the corresponding author, C.K., upon request.

## Appendix A

Have the following laws and regulations facilitated or hindered your innovation over the past three years (2015–2017)? Please evaluate the direction and level of the impact.

**Table A1:** Questionnaire of Korean innovation survey.

Type of Law and Regulation	Promote Innovation		Moderate	Hinder Innovation	
	Significantly	Somewhat	No Effect	Somewhat	Significantly
Occupational (employment and labor) standard and regulation					
